# Engineering Transcriptional Regulation to Control Pdu Microcompartment Formation

**DOI:** 10.1371/journal.pone.0113814

**Published:** 2014-11-26

**Authors:** Edward Y. Kim, Christopher M. Jakobson, Danielle Tullman-Ercek

**Affiliations:** Department of Chemical and Biomolecular Engineering, University of California, Berkeley, California, United States of America; University of Houston, United States of America

## Abstract

Bacterial microcompartments (MCPs) show great promise for the organization of engineered metabolic pathways within the bacterial cytoplasm. This subcellular organelle is composed of a protein shell of 100–200 nm diameter that natively encapsulates multi-enzyme pathways. The high energy cost of synthesizing the thousands of protein subunits required for each MCP demands precise regulation of MCP formation for both native and engineered systems. Here, we study the regulation of the propanediol utilization (Pdu) MCP, for which growth on 1,2-propanediol induces expression of the Pdu operon for the catabolism of 1,2-propanediol. We construct a fluorescence-based transcriptional reporter to investigate the activation of the P_pdu_ promoter, which drives the transcription of 21 *pdu* genes. Guided by this reporter, we find that MCPs can be expressed in strains grown in rich media, provided that glucose is not present. We also characterize the response of the P_pdu_ promoter to a transcriptional activator of the *pdu* operon, PocR, and find PocR to be a necessary component of Pdu MCP formation. Furthermore, we find that MCPs form normally upon the heterologous expression of PocR even in the absence of the natural inducer 1,2-propanediol and in the presence of glucose, and that Pdu MCPs formed in response to heterologous PocR expression can metabolize 1,2-propanediol *in vivo*. We anticipate that this technique of overexpressing a key transcription factor may be used to study and engineer the formation, size, and/or number of MCPs for the Pdu and related MCP systems.

## Introduction

Microcompartments (MCPs) are structures utilized by bacteria to organize and sequester enzymes and the biochemical pathways they catalyze [1]–[3]. Various bacterial MCP systems share a general arrangement of an outer protein shell, made up of thousands of subunits, which contains encapsulated metabolic enzymes within its lumen [4]–[9]. The propanediol utilization (Pdu) MCP, found in several species of enteric bacteria, encapsulates enzymes involved in the metabolism of 1,2-propanediol (1,2-PD) [10]. Twenty-one genes involved in the Pdu pathway are located on the *pdu* operon and are regulated by the P_pdu_ promoter [5], [11]–[14]. The encapsulation of the first few steps in this metabolic pathway sequesters the toxic intermediate propionaldehyde [15], [16]. It is to be noted, however, that synthesizing the thousands of proteins required for Pdu MCP formation comes at a high energy cost. Therefore, regulating the *pdu* operon and limiting MCP formation only to environments containing the substrate 1,2-PD is critical for cell fitness. In fact, due to this requirement for a specific metabolite to form MCPs, the Pdu MCP remained elusive to biologists for many years despite its presence in many well-studied organisms such as *Salmonella enterica*.

In recent years, there has been growing interest in using MCPs as nanobioreactors by encapsulating enzymes for engineered synthetic pathways [14], [17]–[19]. To this end, methods are established for encapsulating heterologous enzymes within MCPs. For instance, in the Pdu system, two of the natively encapsulated enzymes, PduP and PduD, bear N-terminal signal peptides which are sufficient to mediate the encapsulation of heterologous proteins [20], [21]. However, it is also necessary to understand and gain control of the regulatory mechanism that drives MCP formation, potentially enabling the tuning of the timing, copy number, and size of MCPs.

For the Pdu MCP system, previous studies identified the DNA-binding protein PocR to be a *trans*-acting positive regulator of both the *pdu* operon and the adjacent, divergently-transcribed *cob* operon in *S. enterica* [12], [22], [23]. The regulation of these two operons involves five promoters in the *pdu/cob* locus [24]. The substrate 1,2-PD is implicated in an allosteric interaction with PocR leading to activation of the P_cob_ promoter [25]. This allosteric interaction is thought to similarly regulate the P_pdu_ promoter in response to 1,2-PD, in combination with the global Crp and Arc regulatory systems which also affect the level of *pocR* expression [26]. These studies preceded the discovery of the Pdu MCP, and to date the implications of these regulatory mechanisms on MCP expression and formation have not been explored.

Here, we describe the construction and application of a fluorescence-based reporter of transcription from the P_pdu_ promoter to examine the regulation of the Pdu operon with respect to Pdu MCP formation. We first confirm that this transcriptional reporter correlates with MCP formation as assessed by microscopy and biochemical techniques. Using this reporter, we discover that 1,2-PD is sufficient for MCP formation in various rich media, in addition to the previously-reported MCP-inducing NCE minimal media. We then investigate the role of the transcription factor PocR and find it to be a necessary component of the regulation of MCP formation. Furthermore, we find that overexpression of PocR confers MCP formation and function, even in the absence of 1,2-PD and in the presence of glucose, which normally represses expression.

## Materials and Methods

### Bacterial Strains, Media, and Growth Conditions

The bacterial strain used in this study is *Salmonella enterica* serovar Typhimurium LT2. Cultures were grown in 2 mL of LB (lysogeny broth) Miller medium overnight supplemented with the appropriate antibiotic to maintain the plasmid (34 µg/mL chloramphenicol, 50 µg/mL carbenicillin, or 50 µg/mL kanamycin). For growth in rich media, cultures were diluted 1∶100 into lysogeny broth (LB) Miller supplemented with the appropriate antibiotic. For growth in minimal media, cultures were diluted 1∶1000 into no-carbon-E (NCE) minimal medium [27], supplemented with 1 mM MgSO4, 50 µM ferric citrate, half the usual amount of appropriate antibiotic (17 µg/mL chloramphenicol, 25 µg/mL carbenicillin, or 25 µg/mL kanamycin), and 42 mM succinate to support growth in the absence of coenzyme B12 for 1,2-PD metabolism. In cases where MCP formation under natural induction was desired, cultures were supplemented with 55 mM 1,2-PD. For growth on 1,2-PD, overnight cultures in LB Miller were resuspended to OD_600_ = 0.05 in NCE supplemented with 1 mM MgSO4, 50 µM ferric citrate, 55 mM 1,2-PD, and 150 nM coenzyme B12 (adenosylcobalamin) as described previously [28]. OD_600_ was measured subsequently as indicated. Culture volumes were 400 mL of media in 2 L flasks for MCP purification, 10 mL of media in 25 mm by 150 mm culture tubes for growth on 1,2-PD, and 5 mL of media in 24-well blocks (Analytical Sales and Services, Inc., cat. no. 24108) for transcriptional activation experiments.

All cultures were grown at 37°C in an orbital shaker at 225 rpm. For experiments involving gene expression from a plasmid, genes were induced at OD_600_ = 0.4 with 1.33 mM arabinose for expression of PduP^1-18^-GFP from a pBAD33 plasmid, or 1 ng/mL anhydrous tetracycline (aTc) for expression of PocR from a pSC101 pTET plasmid. After five additional hours of growth, samples were taken for fluorescence microscopy, flow cytometry, or Pdu MCP purification.

### Fluorescence microscopy

Bacteria were viewed using a Nikon Ni-U upright microscope with a 100x, 1.45 n.a. plan apochromat objective. Images were captured using an Andor Clara-Lite digital camera. Fluorescence images were collected using a C-FL Endow GFP HYQ band pass filter.

### Pdu MCP purification

The MCP purification protocol was performed by lysis and centrifugation as previously described [29].

### Electron microscopy

10 µL of purified MCPs, at a concentration of 100 µg/mL, were placed on 400 mesh formvar coated copper grids with a carbon film for two minutes. The grids were washed three times with deionized water, then stained with 2% aqueous uranyl acetate for two minutes. Samples were observed and photographed with a Gatan Ultrascan 1000 camera (Gatan, Inc., Pleasanton, CA) on a FEI Tecnai T12 transmission electron microscope.

### Genetic methods

To create the P_pdu_-GFP transcriptional fusion, 373 base pairs upstream (5′) of the *pduA* start codon were cloned from the genome of *S. enterica* serovar Typhimurium LT2 to capture the putative P_pdu_ promoter, and placed into a pPROTET plasmid (Clontech). Downstream (3′) of the P_pdu_ promoter, *gfp* mutant 2 [30], containing its own Shine-Dalgarno sequence and start codon, was inserted to serve as a fluorescent reporter. The P_pdu_ promoter region was PCR amplified from the *S. enterica* serovar Typhimurium LT2 genome using primers CMJ 091 and CMJ 097 (see Table S1 in Material S1), and *gfp* mutant 2 was amplified using primers CMJ 038 and CMJ 096. The vector backbone was PCR amplified from a pPROTET plasmid to introduce BsaI restriction sites using primers CMJ 094 and CMJ 095. These three amplicons were assembled to construct the P_pdu_-GFP transcriptional fusion plasmid by Golden Gate assembly [31].

The *S. enterica* Δ*pocR* knockout strain was constructed using Lambda Red-based recombination as previously described [32]. The primers used for amplification of the kanamycin cassette of pKD13 were EYK 616 and EYK 617.

### Flow cytometry

At the indicated time points, aliquots of each sample were diluted to OD_600_ = 0.01 into 200 µL phosphate-buffered saline (PBS) with 2 mg/mL kanamycin to halt translation. These dilutions were then further diluted 1∶20 into 200 µL of phosphate-buffered saline with 2 mg/mL kanamycin in 96-well plates. The GFP fluorophore was allowed to mature for 30 minutes following dilution of the final time point, and 10,000 events were collected for each sample on a Millipore Guava easyCyte 5HT flow cytometer. Gates were set around the cell population using the forward and side scatter channels, and average population fluorescence values were calculated using the geometric mean. Analysis was performed using FlowJo software (www.FlowJo.com).

### SDS-PAGE and western blotting

Purified MCPs were broken by heating to 95°C in Laemmli buffer and proteins were separated by polyacrylamide gel electrophoresis using 4%–20% (wt./vol.) polyacrylamide gels. GFP was detected by western blotting by standard techniques using mouse anti-GFP primary antibody (Clontech 632375) at a 1∶2000 dilution and horseradish peroxidase-conjugated goat anti-mouse secondary antibody (Thermo Scientific 32430) at a 1∶1000 dilution.

## Results

### Development of a fluorescent reporter for pdu transcription

**Figure 1:**
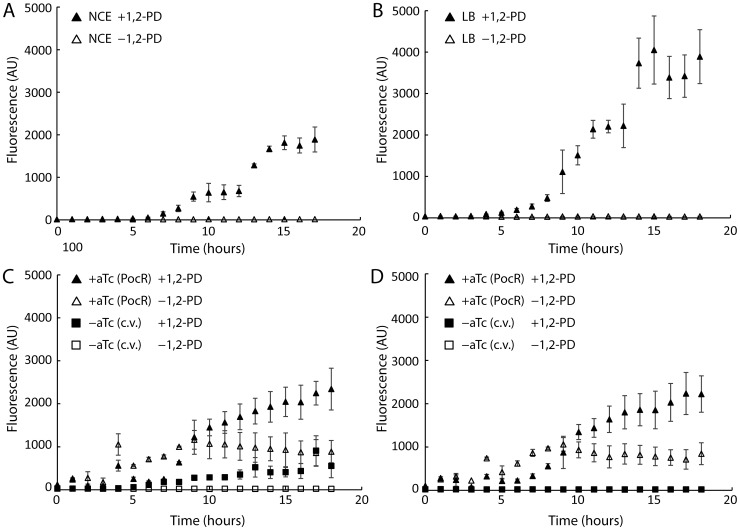
Flow cytometry fluorescence time course of *S. enterica* harboring plasmid P_pdu_–GFP. Time is indicated as hours after OD_600_ = 0.4, at which point cultures continued to grow without 1,2-PD (open symbols) or with the addition of 1,2-PD (solid symbols). (A) Wild-type *S. enterica* grown in NCE minimal media. (B) Wild-type *S. enterica* grown in LB media. (C) Wild-type *S. enterica* grown in LB carrying a secondary plasmid containing either the control vector pTET MBP without aTc (squares), or pTET pocR induced with 1 ng/mL aTc (triangles). (D) *S. enterica* ΔpocR grown in LB carrying a secondary plasmid containing either the control vector pTET MBP without aTc (squares), or pTET pocR induced with 1 ng/mL aTc (triangles).

To quickly assess the impact of various conditions on the regulation of the *pdu* operon, we constructed a reporter plasmid (P_pdu_-GFP) for *pdu* transcription, in which transcription of green fluorescent protein (*gfp*) is driven by activation of the P_pdu_ promoter; this approach was successfully employed to investigate other operons [33], [34]. We first used this reporter to verify that 1,2-PD activates the P_pdu_ promoter in *S. enterica*. Using flow cytometry to measure cellular fluorescence, we observed the first increase in fluorescence two hours after the addition of 1,2-PD (Fig. 1A, see also Fig. S1A in Material S1). Fluorescence continued to increase over the course of several hours, indicating continued transcription from the P_pdu_ promoter.

**Figure 2:**
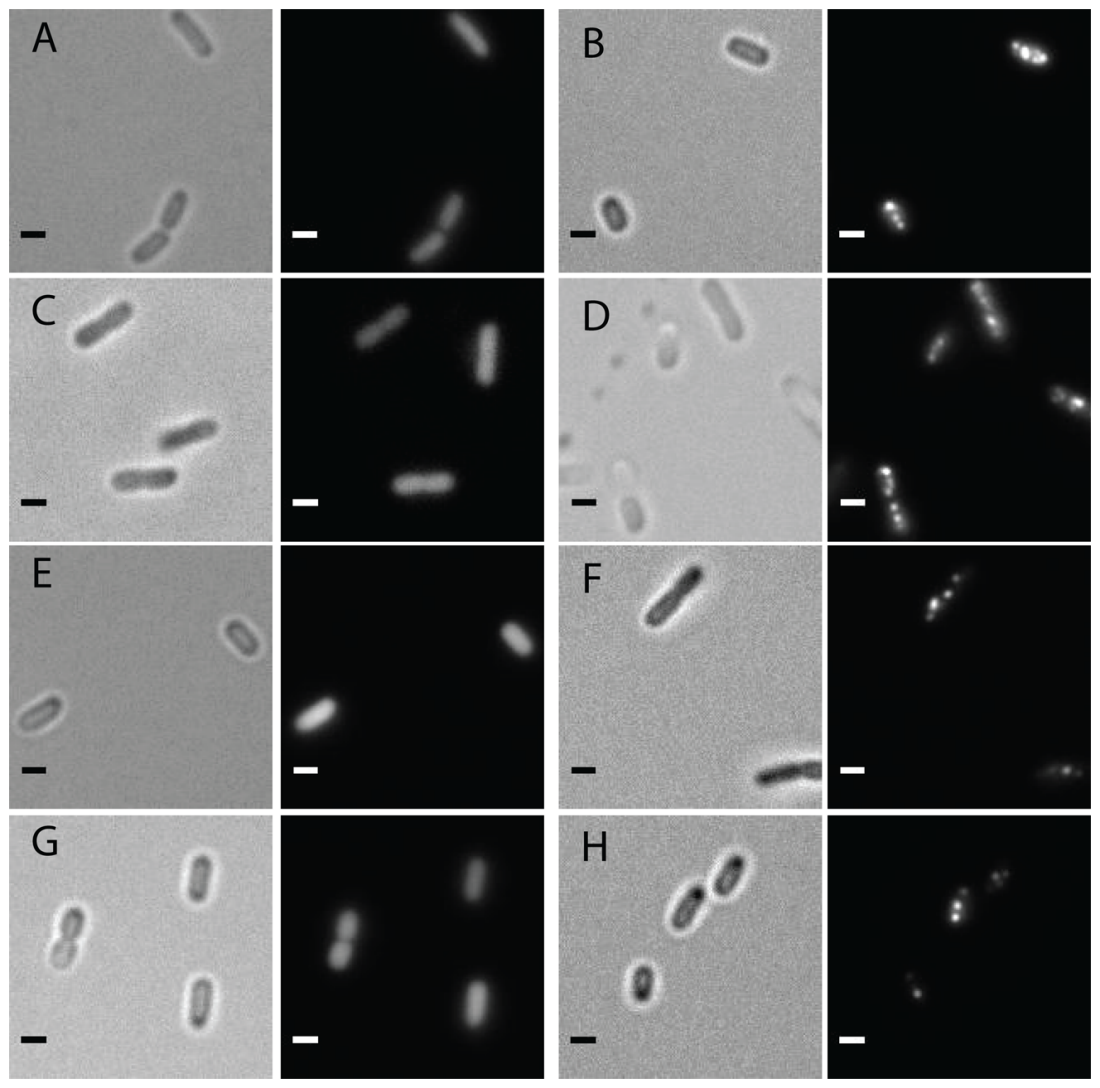
Bright field and fluorescence microscopy of *S. enterica* expressing PduP^1-18^-GFP. Representative images of are shown for *S. enterica* expressing fluorescent encapsulation reporter PduP^1-18^-GFP grown in (A) NCE, (B) NCE 1,2-PD, (C) LB, and (D) LB 1,2-PD. *S. enterica* expressing PduP^1-18^-GFP are grown in LB carrying a secondary plasmid, either (E) the control vector pTET MBP without aTC, or (F) pTET PocR induced with 1 ng/mL aTc. *S. enterica* Δ*pocR* expressing PduP^1-18^-GFP are grown in LB carrying a secondary plasmid containing either (G) the control vector pTET MBP without aTc, or (H) pTET PocR induced with 1 ng/mL aTc. Scale bars represent 1 µm.

We used two methods to verify that activation of the transcriptional reporter correlates with the formation of MCPs. First, we applied another fluorescence-based system in which the first 18 amino acids of the MCP-encapsulated enzyme PduP are fused to GFP to create an MCP-encapsulated fluorescent reporter (Pdu^1-18^-GFP). As previously reported, punctate fluorescence was observed by microscopy when *S. enterica* concurrently express this encapsulation reporter and Pdu MCPs [20], [35] (Fig. 2A, B), indicating localization of the reporter fusion to Pdu MCPs. In a microscopy time course, we first observed cells with one or more fluorescent puncta within two hours after addition of 1,2-PD (2.5% of cells, n = 79), showing agreement with flow cytometry results (see Fig. S2 in Material S1). The proportion of cells with fluorescent puncta increased over time until we observed that nearly all cells contained at least one fluorescent puncta after six hours (95.2% of cells, n = 104).

**Figure 3:**
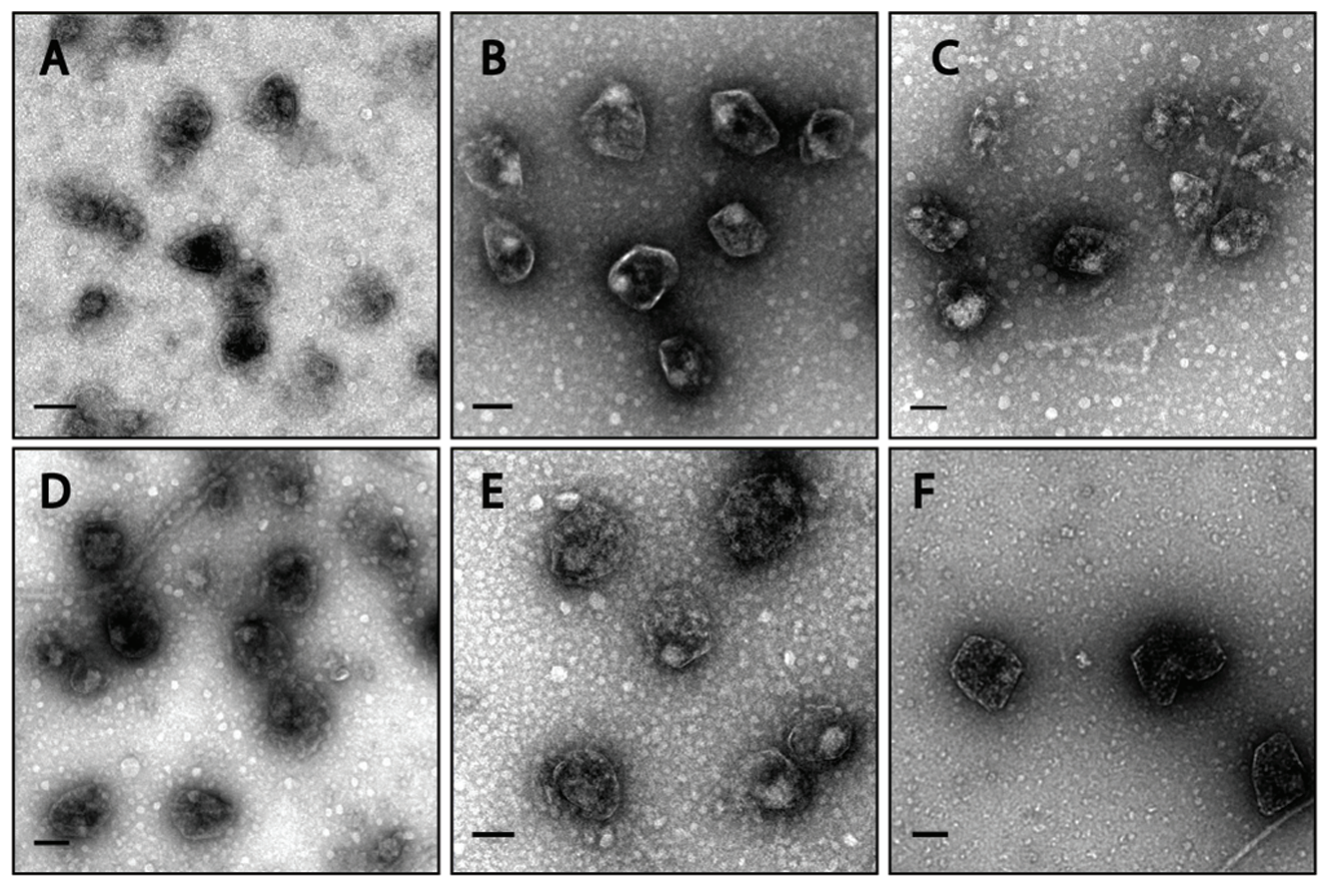
Transmission electron micrographs of purified Pdu MCPs. MCPs are purified from (A) *S. enterica* grown in NCE 1,2-PD, (B) *S. enterica* expressing PduP^1-18^-GFP in NCE 1,2-PD, (C) *S. enterica* expressing PduP^1-18^-GFP in LB 1,2-PD, (D) *S. enterica* co-expressing PduP^1-18^-GFP and PocR in LB, (E) *S. enterica* Δ*pocR* co-expressing PduP^1-18^-GFP and PocR in LB, and (F) *S. enterica* Δ*pocR* co-expressing PduP^1-18^-GFP and PocR in LB with 20 mM glucose. Scale bars represent 100 nm.

**Figure 4:**
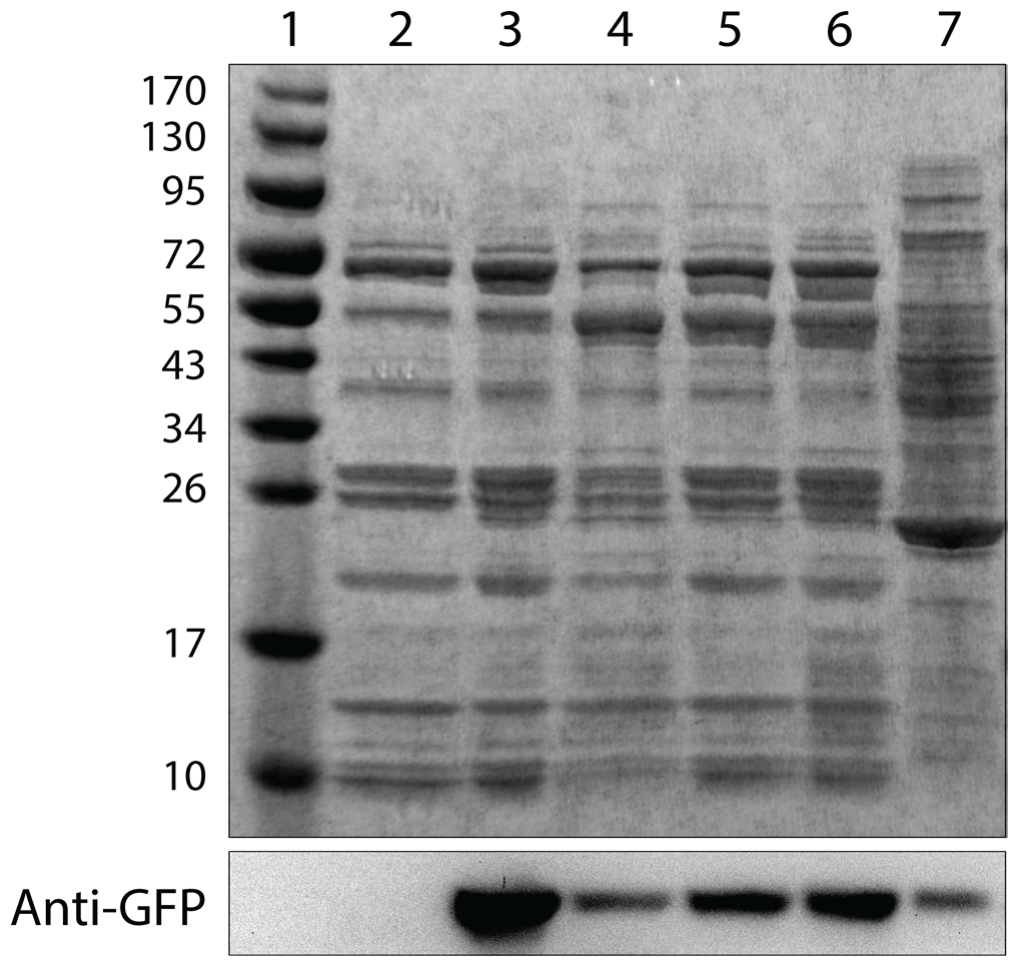
Coomassie-stained gel and western blot of purified MCPs. 4%–20% SDS-PAGE gel stained with Coomassie (top) and anti-GFP western blot (bottom) of a molecular mass marker (lane 1), and purified MCPs from *S. enterica* grown in NCE 1,2-PD (lane 2), *S. enterica* expressing PduP^1-18^-GFP grown in NCE 1,2-PD (lane 3), *S. enterica* expressing PduP^1-18^-GFP grown in LB 1,2-PD (lane 4), *S. enterica* expressing PduP^1-18^-GFP and PocR grown in LB (lane 5), *S. enterica* Δ*pocR* expressing PduP^1-18^-GFP and PocR grown in LB (lane 6), and cell lysate from *S. enterica* expressing PduP^1-18^-GFP grown in LB (lane 7). Lanes with purified MCPs were loaded with 6 µg of total protein.

Next, we purified MCPs from *S. enterica* expressing the encapsulation reporter PduP^1-18^-GFP. When viewed by transmission electron microscopy (TEM), these purified MCPs appeared morphologically similar to purified Pdu MCPs previously reported in literature [10], [20], [29] (Fig. 3A, B). Furthermore, an SDS-PAGE gel of purified MCPs showed a similar banding pattern to those previously reported in literature [10], [20], [29] and an anti-GFP western blot indicated the presence of GFP in purified MCPs (Fig. 4).

### Pdu MCPs form in strains grown in rich media in the absence of glucose

To date, all published work involving endogenous expression of MCPs in *S. enterica* use No-Carbon E (NCE) minimal medium for growth. However, the growth rate of *S. enterica* is much slower in NCE media than in rich media (see Fig. S3 in Material S1), creating additional barriers to effectively using MCPs in research and industry. We investigated whether activation of the P_pdu_ promoter is inhibited in rich media that is not supplemented with glucose using the P_pdu_-GFP transcriptional reporter plasmid, starting with the common growth medium LB. A flow cytometry time course showed increased fluorescence upon addition of 1,2-PD for strains grown in LB (Fig. 1B, see also Fig. S1B in Material S1). This increased fluorescence occurred on a similar time scale when compared to strains grown in NCE 1,2-PD.

Previous studies report that induction of the *pdu* operon requires growth on a poor carbon source because glucose mediates the repression of the P_pdu_ promoter by repressing transcription of P_poc_ via the Crp global regulatory system [24], [26]. We confirmed that the P_pdu_ promoter is not activated in *S. enterica* grown in media containing glucose using flow cytometry with the P_pdu_-GFP transcriptional reporter. *S. enterica* harboring this fluorescent reporter showed no shift in fluorescence upon addition of 1,2-PD when grown in the presence of 20 mM glucose (see Fig. S4 in Material S1).

We again used fluorescence microscopy to verify the formation of MCPs in *S. enterica* expressing the encapsulation reporter PduP^1-18^-GFP. We observed punctate fluorescence for strains grown in LB 1,2-PD similar to the puncta observed in strains grown in NCE 1,2-PD, but not in strains grown in LB without 1,2-PD (Fig. 2C, D). Similar results were seen in strains expressing PduP^1-18^-GFP when grown in the rich media 2xYT and Terrific Broth (see Fig. S5 in Material S1). When viewed by TEM, we observed no apparent morphological differences between MCPs purified from *S. enterica* expressing PduP^1-18^-GFP grown in LB 1,2-PD when compared to MCPs purified from the same strain grown in NCE 1,2-PD (Fig. 3C). A western blot against GFP indicated that purified MCPs from *S. enterica* grown in LB continued to encapsulate heterologous proteins (Fig. 4).

### Pdu MCPs form correctly upon heterologous expression of transcriptional activator PocR

We next set out to use PocR to directly control MCP formation. We tested the effects of PocR on P_pdu_ promoter activation by monitoring fluorescence from the P_pdu_-GFP transcriptional fusion plasmid upon heterologous expression of PocR from a secondary, aTc-inducible pTET-based plasmid. In *S. enterica* grown in LB, expression of PocR by addition of 1 ng/mL aTc resulted in increased fluorescence even in the absence of 1,2-PD, while strains carrying a negative control vector encoding for maltose-binding protein (MBP) showed no shift in fluorescence (Fig. 1C, see also Fig. S6 in Material S1). When PocR was overexpressed in combination with the addition of 1,2-PD, we observed a higher fluorescence shift than induction by either 1,2-PD or PocR expression alone (Fig. 1C, see also Fig. S6 in Material S1). Fluorescence microscopy of strains co-expressing PocR and encapsulation reporter PduP^1-18^-GFP showed punctate fluorescence, while strains expressing PduP^1-18^-GFP and carrying the MBP control vector showed diffuse fluorescence (Fig. 2E, F). In the case of *S. enterica* grown in the presence of glucose, we observed that heterologous expression of PocR restored activation of the P_Pdu_ promoter both in the presence and absence of 1,2-PD (see Fig. S4 in Material S1). In this case, fluorescence microscopy could not be used to track MCP formation for strains expressing PduP^1-18^-GFP due to catabolite repression by glucose of the pBAD induction system.

To confirm previous reports [36] that PocR is a necessary component of Pdu MCP regulation, we generated a *pocR* knockout strain in *S. enterica* using Lambda Red-based recombination. In *S. enterica* Δ*pocR* harboring P_pdu_-GFP, the addition of 1,2-PD no longer resulted in a shift in fluorescence when measured by flow cytometry (Fig. 1D, see also Fig. S7 in Material S1). Furthermore, fluorescence microscopy of *S. enterica* Δ*pocR* expressing PduP^1-18^-GFP in the presence of 1,2-PD displayed diffuse fluorescence, indicating that the fusion protein is not localized in the absence of MCPs (Fig. 2G).

We next used *S. enterica* Δ*pocR* harboring P_pdu_-GFP to demonstrate that expression of PocR from an inducible plasmid can complement the genomic disruption. Indeed, we observed a shift in fluorescence both in the presence and absence of 1,2-PD when PocR expression is induced, as measured by flow cytometry (Fig. 1D, see also Fig. S7 in Material S1). Fluorescence microscopy showed that the punctate fluorescence phenotype is restored for *S. enterica* Δ*pocR* expressing PduP^1-18^-GFP and PocR even in the absence of 1,2-PD (Fig. 2H). TEM images of purified MCPs from strains expressing PocR in the absence of 1,2-PD showed no apparent morphological differences, for both wild type *S. enterica* and *S. enterica* Δ*pocR*, when compared to MCPs purified from strains induced by 1,2-PD (Fig. 3D, E). TEM images of MCPs purified from a *pocR* knockout strain of *S. enterica* expressing PocR in the presence of 20 mM glucose and in the absence of 1,2-PD also appeared morphologically normal (Fig. 3F). A western blot against GFP indicates that purified MCPs from *S. enterica* induced by PocR overexpression in the absence of 1,2-PD encapsulated heterologous proteins (Fig. 4).

### Pdu MCPs formed upon heterologous expression of transcriptional activator PocR metabolize 1,2-PD *in vivo*

We finally tested whether the Pdu MCPs formed following heterologous PocR expression were functional for 1,2-PD metabolism. While a *pocR* knockout strain of *S. enterica* expressing MBP from a control vector showed no growth in media containing 1,2-PD as the sole carbon source, growth on 1,2-PD was restored in a *pocR* knockout strain over-expressing PocR. Furthermore, *S. enterica* Δ*pocR* over-expressing PocR grew faster than wild type *S. enterica* expressing MBP from a control vector, which only expresses native levels of PocR from its genomic locus. This increase in growth rate is also observed for wild type *S. enterica* over-expressing PocR. (see Fig. S8 in Material S1).

## Discussion

The control of *S. enterica* Pdu MCP formation is an important step towards engineering MCP-based biotechnological tools. To aid in characterizing Pdu MCP regulation, we constructed a fluorescence-based transcriptional reporter plasmid to measure activation of the P_pdu_ promoter, and use this technique in conjunction with fluorescence microscopy and TEM to investigate the regulation that underlies MCP formation.

First, we find that *S. enterica* may be grown in rich media without inhibiting MCP formation, despite suggestions in the literature that growth on a poor carbon source is required [24], [26]. While we observe that glucose inhibits MCP expression, this inhibition can be circumvented by overexpression of the transcription factor PocR, supporting previous studies that indicated the global Crp regulatory mechanism acts on P_poc_ rather than directly on P_pdu_ [26]. The ability to form MCPs in a variety of media may prove useful for the biotechnology community, as the growth rate of *S. enterica* is higher in many common rich media when compared to NCE minimal media.

Next, we show that the transcription factor PocR is a necessary component of MCP regulation, and heterologous expression of PocR in the absence of 1,2-PD results in the formation of MCPs morphologically similar to those formed upon induction by 1,2-PD. We further find that Pdu MCPs expressed in the absence of chromosomal *pocR* are functional for 1,2-PD metabolism *in vivo*, suggesting that these MCPs are encapsulating the appropriate Pdu metabolic enzymes in addition to forming morphologically normal MCPs. In fact, PocR overexpression led to faster growth even in a *S. enterica* strain with intact chromosomal *pocR*. This could be due to earlier Pdu MCP formation, as a result of apparently earlier transcriptional activation as observed using the transcriptional fusion, or another factor such as different enzyme loading to the MCPs or different numbers of MCPs per cell with respect to the wild type.

While an allosteric interaction between PocR and 1,2-PD is thought to be required for P_pdu_ activation at native expression levels of PocR, high levels of PocR by heterologous overexpression appear to be sufficient to overcome the requirement of 1,2-PD for PocR binding, and still result in normal MCP formation. Supporting this theory, we observe higher levels of fluorescence from the P_pdu_–GFP reporter when both PocR is overexpressed and 1,2-PD is present compared to PocR overexpression alone, consistent with an allosteric interaction increasing the affinity of PocR for its target sequence.

Further characterization of PocR using the methods described in this paper may prove to be informative, not only in further elucidation of the regulatory mechanism behind MCP formation, but also toward creating novel MCP expression phenotypes. For example, while in our studies we use a low concentration of aTc to induce PocR expression, we speculate that *pocR* expression levels can be modulated to either change the average number of MCPs formed per cell or vary the size of the MCPs, both of which would be of interest for utilizing the Pdu MCP as a nanobioreactor. It is also desirable to develop methods of controlling MCP expression and formation in a biotechnological context, absent its native regulatory molecule 1,2-PD. To our surprise, the Pdu MCPs formed by PocR overexpression are morphologically normal and indistinguishable from those formed in response to 1,2-PD. The robust formation of MCPs in response to different transcriptional dynamics, in the case of PocR overexpression, than in the native context raises questions as to the mechanism governing their formation. Further studies are required to determine which aspects of P_pdu_ transcriptional regulation are important in regulating these properties. The insights gained from the regulation of the Pdu MCP are likely generalizable to other MCPs, and may lead to the discovery of new MCP systems. While genetic analysis predicts the existence of many different MCP systems, only a few—most notably the carboxysome, Eut, and Pdu MCPs—were discovered experimentally, likely due to the requirement of a specific metabolite for expression. Many other systems are predicted using bioinformatics, and for one system a protein shell has even been generated in *Escherichia coli* [37], but the shell structures and functions in the native hosts are still largely unknown. The approaches described herein provide two high-throughput alternatives to screening a library of metabolites for MCP formation by low-throughput techniques such as TEM. One approach is to search bacterial genomes for operons containing MCP shell homologs accompanied by homologs of the positive regulator PocR. These MCPs may then be expressed and characterized via the heterologous expression of their regulatory protein alone, in the absence of their unknown natively-inducing metabolite(s). Alternatively, a reporter of the activity of the putative promoter region of the homologous operon, analogous to the transcriptional fusion used in this study, can be screened by flow cytometry or another high-throughput technique for activation in response to a library of metabolites. Promising candidates can then be further screened for MCP formation.

It is surprising that morphologically normal compartments, which require a set number of shell-forming proteins, can form even when concentrations of the shell-forming proteins depart far from native levels. Studies of other MCP systems will shed light on the robust mechanism of MCP formation observed here. The Eut MCP, for example, is regulated by a positive transcriptional regulator EutR that is transcribed at the 3′ end of the Eut operonpolycistron, instead of divergently transcribed on another cistron as is PocR. If other MCPs also form morphologically normal compartments in response to overexpression of their transcriptional regulators, this would suggest that post-transcriptional regulation is important to the assembly mechanism. We show that the combination of measuring transcriptional output and phenotypic observations is a powerful approach for characterizing gene regulation. The results presented in this paper provide further insight into the natural regulation of Pdu MCP formation in *S. enterica*, and are an important step towards utilizing MCPs as a biotechnological tool.

## Supporting Information

Material S1 **Supporting figures and tables.** Table S1. Primers used in this study. Figure S1. Flow cytometry green fluorescence histograms of *S. enterica* harboring plasmid P_pdu_–GFP. Figure S2. Bright field and fluorescence microscopy time course of *S. enterica* after induction with 1,2-PD. Figure S3. Growth curves of *S. enterica* grown in various media. Figure S4. Flow cytometry green fluorescence histograms of *S. enterica* harboring plasmid P_pdu_–GFP. Figure S5: Bright field and fluorescence microscopy of *S. enterica* expressing PduP^1-18^-GFP in 2xYT and Terrific Broth. Figure S6. Flow cytometry green fluorescence histograms of *S. enterica* harboring plasmid P_pdu_–GFP. Figure S7. Flow cytometry green fluorescence histograms of *S. enterica* harboring plasmid P_pdu_–GFP. Figure S8: Growth curves of *S. enterica* grown on 1,2-PD.
(DOCX)
