# Supplementary material for: Engineering Transcriptional Regulation to Control Pdu Microcompartment Formation
Source: PLoS One. 2014 Nov 26;9(11):e113814. doi: 10.1371/journal.pone.0113814 (PMC4245221; doi:10.1371/journal.pone.0113814)
Supplement: Material S1 — Supporting figures and tables. Table S1. Primers used in this study. Figure S1. Flow cytometry green fluorescence histograms of S. enterica harboring plasmid Ppdu–GFP. Figure S2. Bright field and fluorescence microscopy time course of S. enterica after induction with 1,2-PD. Figure S3. Growth curves of S. enterica grown in various media. Figure S4. Flow cytometry green fluorescence histograms of S. enterica harboring plasmid Ppdu–GFP. Figure S5: Bright field and fluorescence microscopy of S. enterica expressing PduP1-18-GFP in 2xYT and Terrific Broth. Figure S6. Flow cytometry green fluorescence histograms of S. enterica harboring plasmid Ppdu–GFP. Figure S7. Flow cytometry green fluorescence histograms of S. enterica harboring plasmid Ppdu–GFP. Figure S8: Growth curves of S. enterica grown on 1,2-PD. (DOCX) [file pone.0113814.s001.docx]

**Supplemental Material**

**Engineering transcriptional regulation to control Pdu microcompartment formation**

Edward Y. Kim, Christopher M. Jakobson, and Danielle Tullman-Ercek

**Table S1. Primers used in this study**

| **Primer name** | **Sequence** |
| --- | --- |
|  |  |
| **CMJ 091** | ATTGGTCTCACATGTTTTGCTTTTCCGATATTTTTAGCAGCCCA |
| **CMJ 107** | ATTGGTCTCATCTAGAAAAGACCTCGCATGGAGTGTTCC |
| **CMJ 038** | ATTGGTCTCATAGATTAAAGAGGAGAAAGGTCATGAGTAAAGGAGAAGAACTTTTCACTGGAG |
| **CMJ 096** | ATTGGTCTCATTTATTTGTATAGTTCATCCATGCCATGTG |
| **CMJ 094** | ATTGGTCTCATAAAGATCTAAAGCTAGAGGCATCAAATAAAAC |
| **CMJ 095** | ATTGGTCTCACATGCTCGAGGTGAAGACGAAAGG |
| **EYK 616** | CAGGGCCAGGATAATGGTAATAAAAAGTGAATGTAAATAATGTGTTTTGTTTATAACAATAAATTAACGTGTAGGCTGGAGCTGCTTC |
| **EYK 617** | ATTTTCTTTCTGAAATGATTAAGATACAAAAGACTATCAAAAATCGGCAATAGCAAAATATTGCTATATTCCGGGGATCCGTCGAC |

**Figure S1. Flow cytometry green fluorescence histograms of *S. enterica* harboring plasmid P_pdu_–GFP.** Time is indicated as hours after OD_600_=0.4, at which point cultures continued to grow with or without 1,2-PD or aTc. (A) Wild type *S. enterica* grown in NCE without 1,2-PD (dashed line) or with 1,2-PD (solid line). (B) Wild type *S. enterica* grown in LB Miller without 1,2-PD (dashed line) or with 1,2-PD (solid line).


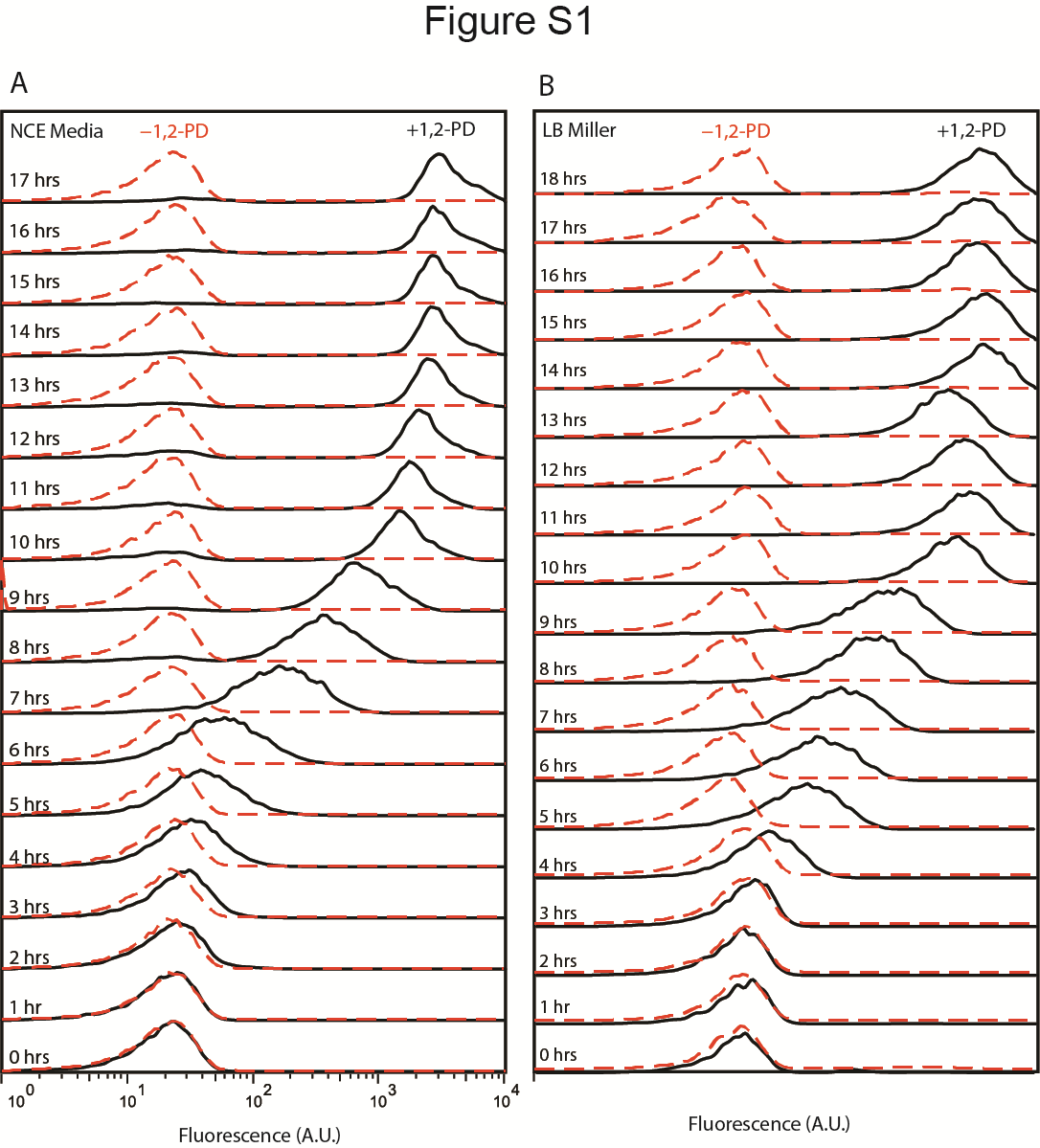

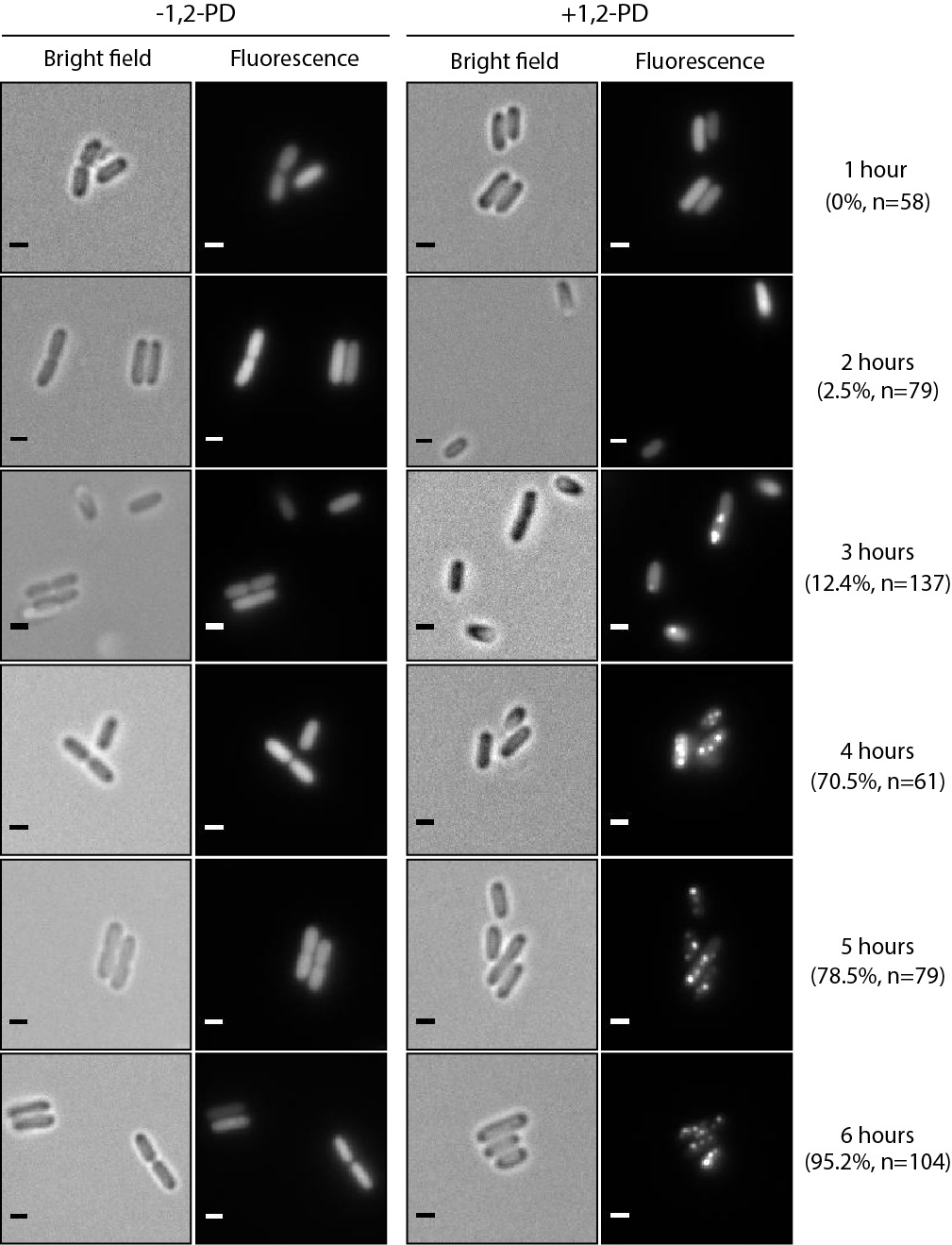


**Figure S2. Bright field and fluorescence microscopy time course of *S. enterica* after induction with 1,2-PD.** *S. enterica*, expressing the encapsulation reporter PduP^1-18^-GFP, are grown in NCE minimal media. Time is measured in hours after reaching OD_600_=0.4, at which point cultures were induced with 0.02% arabinose to express the encapsulation reporter PduP^1-18^-GFP, and 0.4% 1,2-PD to express the Pdu operon. For cultures grown with 1,2-PD, the percentage of cells containing one or more fluorescent puncta is shown. Scale bars represent 1 μm.


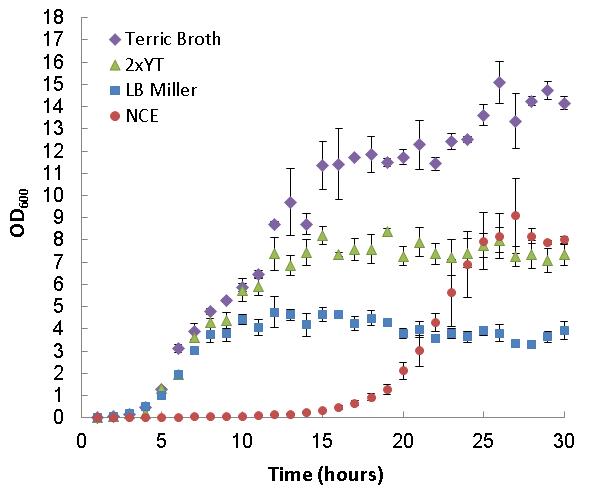


Figure S3. Growth curves of *S. enterica* grown in various media. Overnight cultures of *S. enterica* were diluted to an initial OD_600_=0.01 in Terrific Broth (diamonds), 2xYT (triangles), LB Miller (squares), and NCE media (circles) (n=3). Error bars represent one standard deviation.


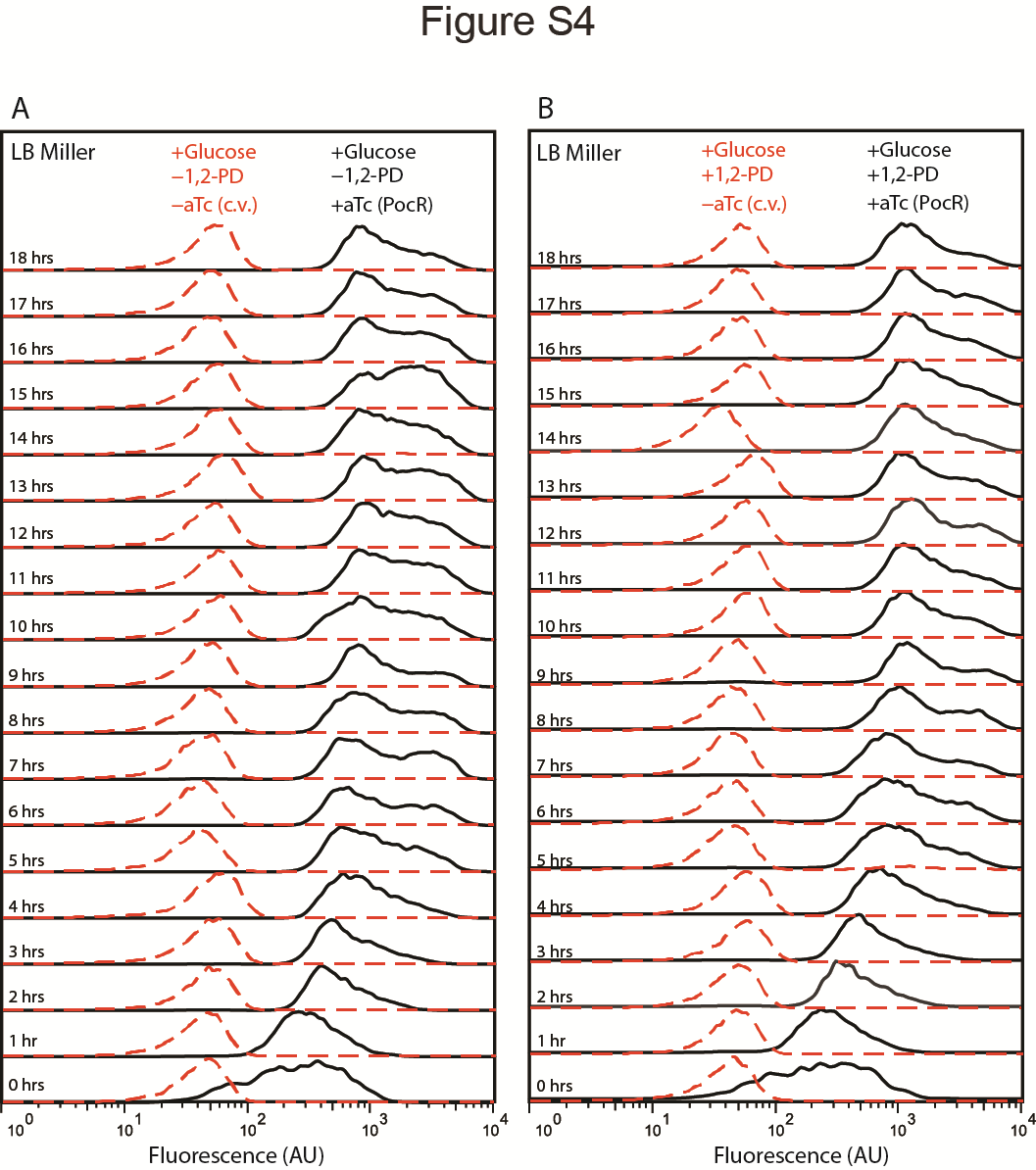


**Figure S4. Flow cytometry green fluorescence histograms of *S. enterica* harboring plasmid P_pdu_–GFP.** Wild type *S. enterica* grown in LB Miller supplemented with 20 mM glucose without 1,2-PD (A), and with 1,2-PD (B). Strains carry an additional plasmid, either the control vector pTET MBP with no aTc (dashed line), or pTET PocR with 1 ng/ml aTc (solid line).


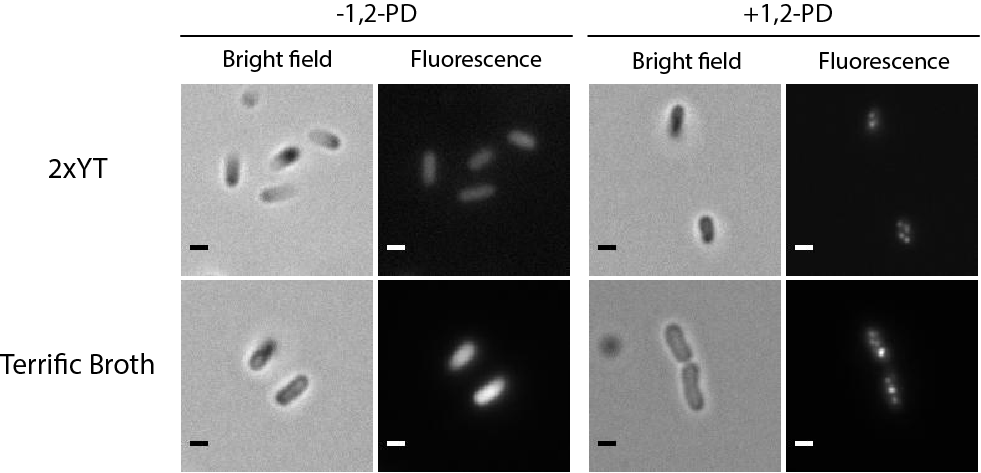


**Figure S5: Bright field and fluorescence microscopy of *S. enterica* expressing PduP^1-18^-GFP in 2xYT and Terrific Broth.** *S. enterica*, expressing the encapsulation reporter PduP^1-18^-GFP, are grown in either 2xYT or Terrific broth media. When cultures reach OD_600_=0.4, they are induced with 0.02% arabinose to express the encapsulation reporter PduP^1-18^-GFP, and 0.4% 1,2-PD to express the Pdu operon if indicated. Images are taken five hours after induction. Scale bars represent 1 μm.


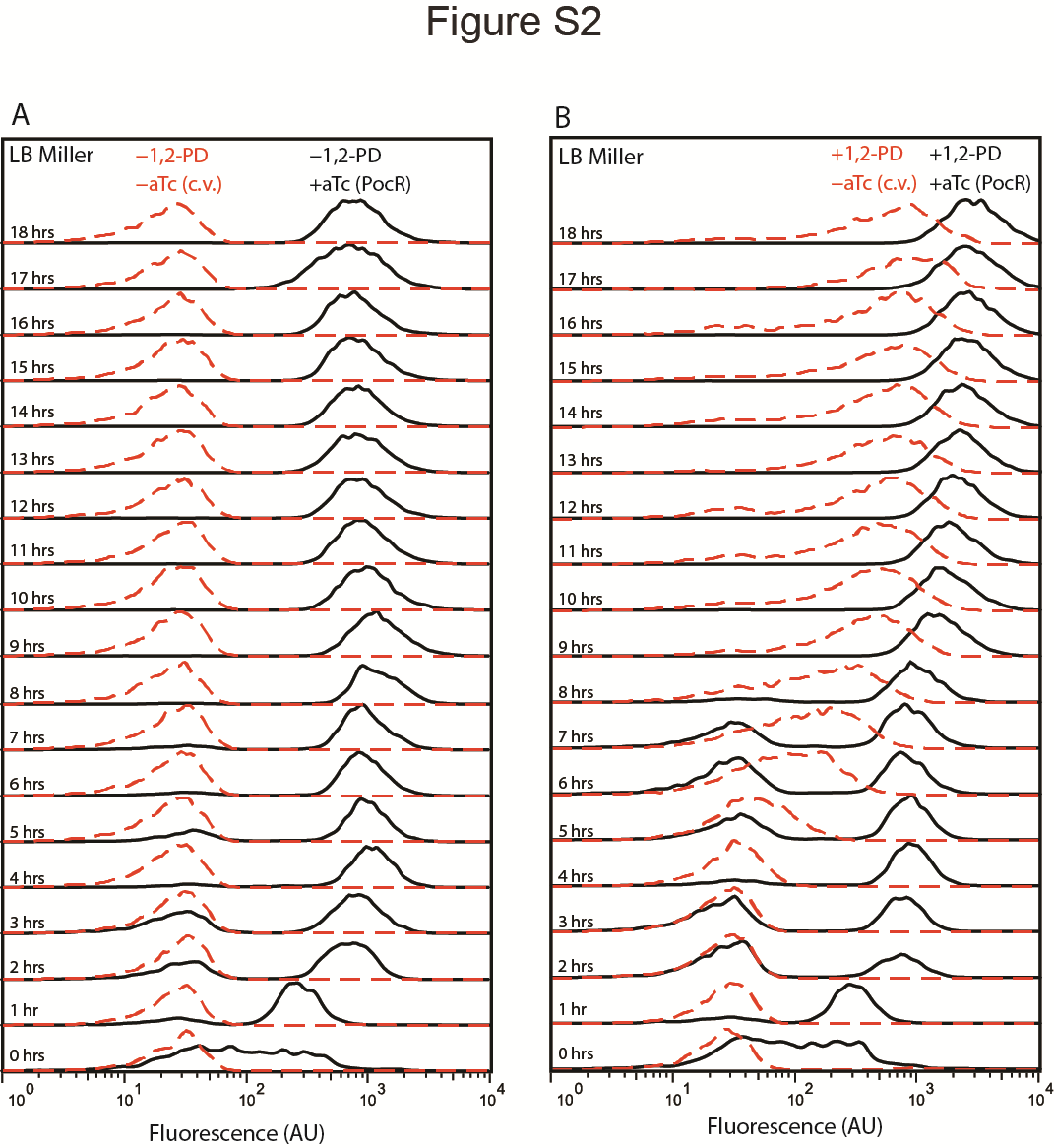


**Figure S6. Flow cytometry green fluorescence histograms of *S. enterica* harboring plasmid P_pdu_–GFP.** Wild type *S. enterica* grown in LB Miller without 1,2-PD (A), and with 1,2-PD (B). Strains carry an additional plasmid, either the control vector pTET MBP with no aTc (dashed line), or pTET PocR with 1 ng/ml aTc (solid line).


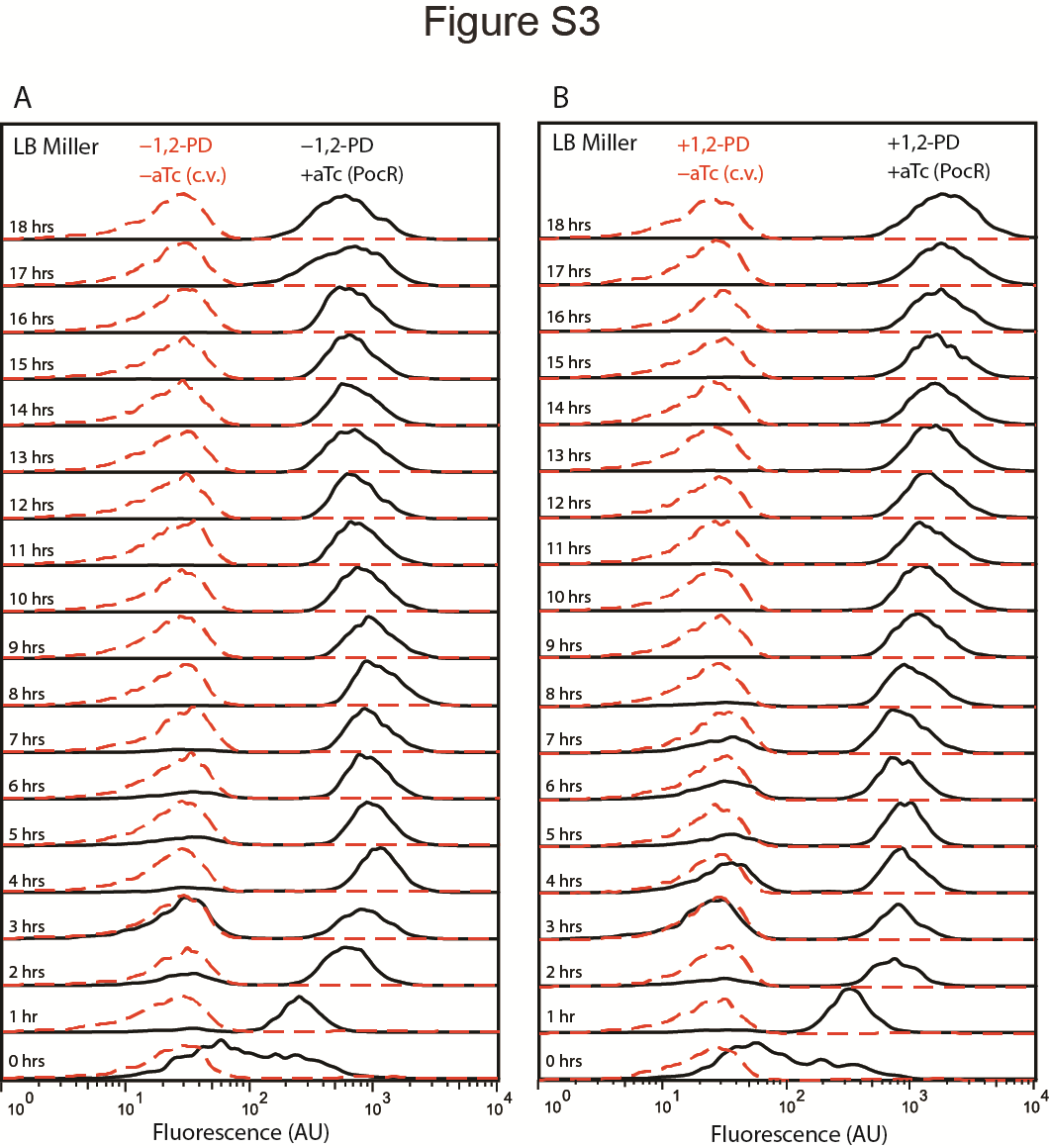


**Figure S7. Flow cytometry green fluorescence histograms of *S. enterica* harboring plasmid P_pdu_–GFP.** *S. enterica* Δ*pocR* grown in LB Miller without 1,2-PD (A), and with 1,2-PD (B). Strains carry an additional plasmid, either the control vector pTET MBP with no aTc (dashed line), or pTET PocR with 1 ng/ml aTc (solid line).


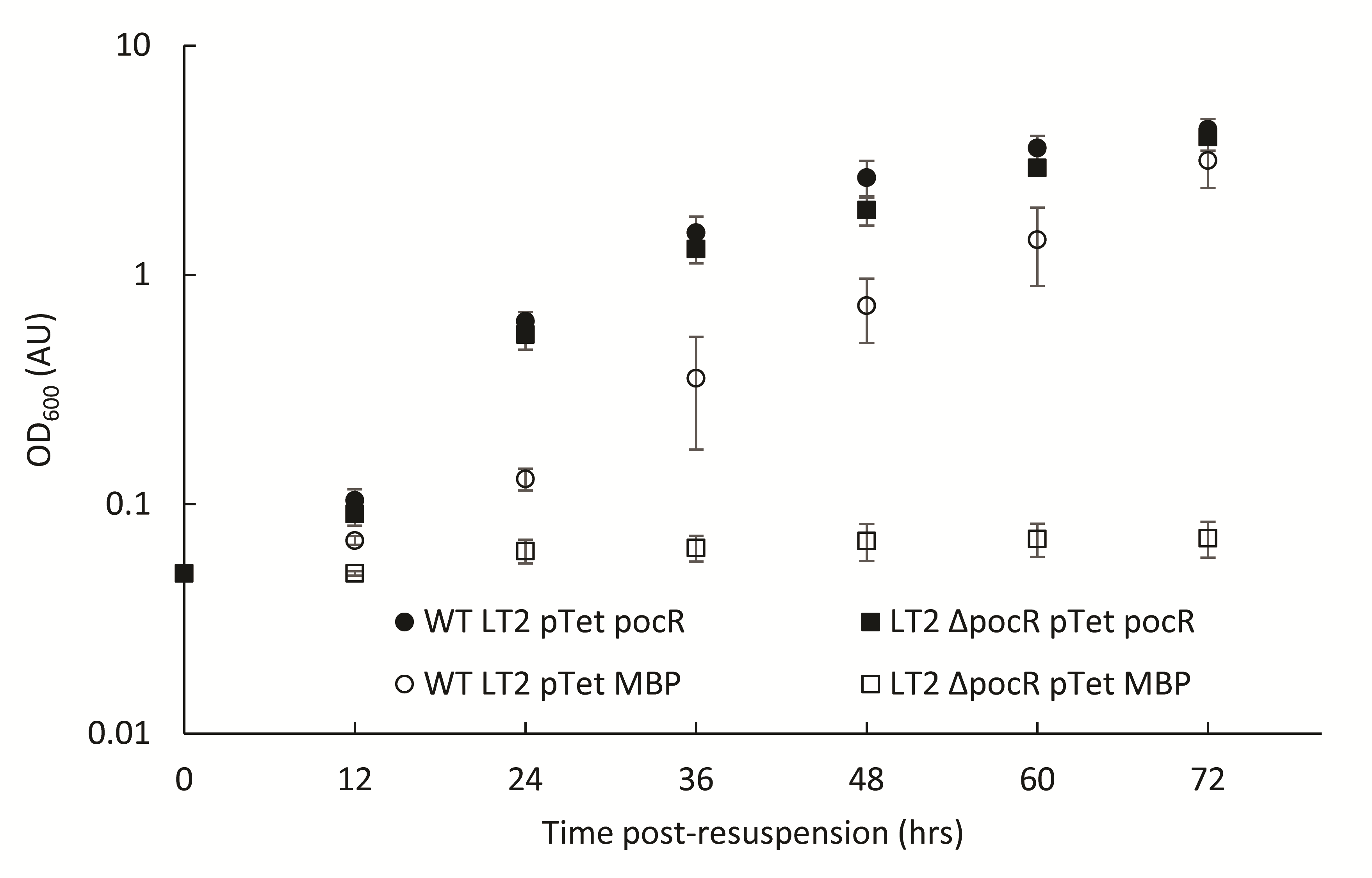


**Figure S8: Growth curves of *S. enterica* grown on 1,2-PD.** Wild type *S. enterica* (circles) and *S. enterica* Δ*pocR* (squares) bearing an additional plasmid, either the control vector pTET MBP (open symbols), or pTET PocR (solid symbols), each with 1 ng/ml aTc added at time of resuspension. Overnight cultures in LB Miller were resuspended to OD_600_=0.05 in NCE with 55 mM 1,2-PD and 150 nM coenzyme B12 and grown for 72 hours. Data shown are the mean of three independent replicates; error bars indicate one standard deviation.
